# Violence against primary school children with disabilities in Uganda: a cross-sectional study

**DOI:** 10.1186/1471-2458-14-1017

**Published:** 2014-09-29

**Authors:** Karen M Devries, Nambusi Kyegombe, Maria Zuurmond, Jenny Parkes, Jennifer C Child, Eddy J Walakira, Dipak Naker

**Affiliations:** London School of Hygiene & Tropical Medicine, 15-17 Tavistock Place, London, WC1H 9SH UK; Institute of Education, London, UK; Makerere University, Kampala, Uganda; Raising Voices, Kampala, Uganda

**Keywords:** Disability, Children, Adolescents, School, Violence, Uganda

## Abstract

**Background:**

150 million children live with disabilities globally, and a recent systematic review found 3 to 4 times the levels of violence versus non-disabled children in high income countries. However, almost nothing is known about violence against disabled children in lower income countries. We aim to explore the prevalence, patterns and risk factors for physical, sexual and emotional violence among disabled children attending primary school in Luwero District, Uganda.

**Methods:**

We performed a secondary analysis of data from the baseline survey of the Good Schools Study. 3706 children and young adolescents aged 11-14 were randomly sampled from 42 primary schools. Descriptive statistics were computed and logistic regression models fitted.

**Results:**

8.8% of boys and 7.6% of girls reported a disability. Levels of violence against both disabled and non-disabled children were extremely high. Disabled girls report slightly more physical (99.1% vs 94.6%, p = 0.010) and considerably more sexual violence (23.6% vs 12.3%, p = 0.002) than non-disabled girls; for disabled and non-disabled boys, levels are not statistically different. The school environment is one of the main venues at which violence is occurring, but patterns differ by sex. Risk factors for violence are similar between disabled and non-disabled students.

**Conclusions:**

In Uganda, disabled girls are at particular risk of violence, notably sexual violence. Schools may be a promising venue for intervention delivery. Further research on the epidemiology and prevention of violence against disabled and non-disabled children in low income countries is urgently needed.

## Background

Globally, 150 million children aged 0-18 are estimated to be living with a disability, the majority of whom live in low and middle-income countries (LMICs) [1]. The Convention on the Rights of the Child, and the Convention on the Rights of Persons with Disabilities, direct governments to ensure all children, irrespective of any disability, enjoy their rights without discrimination [2, 3]. Despite these commitments, a growing body of evidence indicates that disabled children are often amongst the most socially excluded and vulnerable [1].

Violence is both a key risk factor for, and consequence of, social exclusion and vulnerability. Evidence from a recent systematic review indicates that children with disabilities are three to four times more likely to be victims of violence than their peers without disabilities [4]. This review drew on studies conducted in high income countries [4]. The majority of evidence from LMICs consists of small-scale qualitative studies by the United Nations and non-governmental organisations [5–8]. This qualitative research has identified multiple forms of discrimination faced by children with disabilities, including stigma, denial of access to school, lack of support for special educational needs and reduced employment prospects [9–11]. Poverty, gender inequalities and disability have been shown to interact to increase the risk of violence and discrimination against children in some contexts, suggesting that disabled girls might be at particular risk [12]. However, existing studies generally do not explore differences by sex, or collect information on who the perpetrators of violence are—a recent systematic review found that the perpetrator was stated in only 12 of 17 included studies [4].

In many settings, disabled children are less likely to be attending school, and have lower levels of educational attainment than their peers [13]. Understanding barriers to school attendance and performance is crucial for understanding how to improve the experience of disabled children in schools in LMICs. To our knowledge, virtually no evidence is available concerning the experience of disabled schoolchildren in Uganda. However, violence against the general population of Ugandan schoolchildren is common, and both demographic and mental health difficulties are associated with increased risk of experiencing violence [14]. Based on previous research, we hypothesized that for disabled children, violence would be more common than among the general population. Drawing on recent empirical findings around the multiplicative effects of multiple vulnerabilities on poor health outcomes [15], we also hypothesised that there could be an interaction effect, where the presence of demographic and mental health risk factors in disabled children might confer even more risk of violence than they do in the general population.

The present study was conducted in the Luwero District which has a mixture of urban and rural communities and demographic characteristics that are broadly similar to the rest of Uganda. 7.1% of Uganda’s population is estimated to have a disability, equivalent to approximately 2.1 million people, with children accounting for 31% of the total [16]. Uganda is generally considered progressive in terms of policies related to disability and has a National Policy on Disability (2005), which “aims at promoting equal opportunities for enhanced empowerment, participation and protection of rights of people with disabilities irrespective of gender, age and type of disability” [17]. The Ministry of Education and Sports also makes reference to a draft Special Needs and Inclusive Education Policy (2011) which aims to encourage increased enrolment, participation and completion of schooling by children with special learning needs [18].

In this paper, we provide data on the epidemiology of violence against disabled children and young adolescents attending primary school in Luwero District. Disability is defined through children’s self-accounts, and includes domains of sight, hearing, mobility, and speech. For disabled students, we sought to 1) describe the prevalence of different types of violence, (physical, sexual, emotional violence and neglect), and the extent to which individual children may experience multiple forms of violence; 2) describe the most common perpetrators of violence; and 3) to explore whether the association between increased risk of violence and mental health difficulties and other selected factors differed between disabled and non-disabled students.

## Methods

We use data from The Good Schools Study [19], a cluster randomised controlled trial of a school-based intervention by Ugandan NGO Raising Voices, designed to prevent violence against children and improve educational outcomes (http://raisingvoices.org/good-school/). The baseline survey was conducted between June and July 2012. Full ethical approvals were given by the London School of Hygiene and Tropical Medicine and the Uganda National Council of Science and Technology. Results for the general population of Luwero school children are reported elsewhere [14].

### Sampling

Based on the most up-to-date records of the Ministry of Education and Sports, 268 schools were operating in Luwero in 2010. Owing to their small size (having less than 40 registered students in Primary 5) 97 schools plus a further 20 schools with existing governance interventions were excluded. The remaining 151 schools constituted our sampling frame. The 151 schools were stratified according to the gender ratio of pupils (>60% girls, >60% boys or approximately even). 42 schools were randomly selected, proportional to the size of the stratum. All schools that were approached agreed to participate. The sampled schools represent 79.7% of Primary (P) 5, 6, and 7 students in the District. A simple random sample of up to 130 pupils across P5, P6 and P7 was taken within each school. In schools that had fewer than 130 students in the target years, all students were invited to participate. Data were obtained from 77% of sampled students. 19% of students were absent from school during the week of data collection or for extended periods and the remaining 4% either refused, were ineligible or had a parent opt them out.

### Procedure

Staff, students and parents from each participating school were notified in advance of the survey by the head teacher. Parents could choose to opt their child out of participation, but there was no requirement for full parental consent in this study. Instead, individual children provided full written consent to participate. Data were collected in private face-to-face interviews. Survey interviewers received three weeks of specialised training on violence research including how to ask questions in a non-judgemental way, preserve confidentiality and on procedures to follow if participants became distressed. In conjunction with local services, the study team also developed a comprehensive child protection plan to provide support to those that were identified to be in need of services. A trained counsellor was available for any child who requested counselling. The response of services is the subject of a separate paper [20].

### Instruments

All survey tools and instruments were reviewed by a panel comprising teachers and Raising Voices staff to ensure their contextual appropriateness. They were then translated into Luganda and iteratively refined in a sample of approximately 40 children from primary schools in Kampala to ensure that they were cognitively accessible and that the meanings of original items were adequately captured. Following this, a larger sample of 697 students and 40 staff from Kampala schools were surveyed to test study procedures and the distribution of items.

Disability was measured using a single question with multiple response options, and included domains of sight, hearing, mobility, speech and whether or not students had epilepsy (Table 1). It was modelled as a binary variable. This method of assessing disability focuses on functional limitations.

**Table 1:** **Definitions of key variables**

Variable name	Items	Coding
Disabled	Do you have any mental or physical disability? For example, do you have trouble seeing, walking, speaking, fits, or anything else? Response options: None, Trouble seeing, Trouble hearing, Trouble walking/with movement, Trouble with speech, Fits, Other.	Coded 1 if answered yes to any of the items; 0 if answered no to all items.
Emotional violence, neglect	Cursed, insulted, shouted at or humiliated you? Referred to your skin colour/gender/religion/tribe or health problems you have in a hurtful way? Stopped you from being with other children to make you feel bad or lonely? Tried to embarrass you because you were an orphan or without a parent? Embarrassed you because you were unable to buy things? Stole or broke or ruined your belongings? Threatened you with bad marks that you didn’t deserve? Accused you of witchcraft?	Coded 1 if answered yes to any of the items; 0 if answered no to all items.
Physical violence	Hurt you or caused pain to you? Slapped you with a hand on your face or head as punishment? Slapped you with a hand on your arm or hand? Twisted your ear as punishment? Twisted your arm as punishment? Pulled your hair as punishment? Hit you by throwing an object at you? Hit you with a closed fist? Hit you with a stick? Caned you? Kicked you? Knocked you on the head as punishment? Made you dig, slash a field, or do other labour as punishment? Hit your fingers or hands with an object as punishment? Crushed your fingers or hands as punishment? Made you stand/kneel in a way that hurts to punish you? Made you stay outside for example in the heat or rain to punish you? Burnt you as punishment*? Taken your food away from you as punishment? Forced you to do something that was dangerous? Choked you*? Tied you up with a rope or belt at school? Tried to cut you purposefully with a sharp object*? Severely beat you up*?	Coded 1 if answered yes to any of the items; 0 if answered no to all items.
*severe physical violence		
Sexual violence	Teased you or made sexual comments about your breasts, genitals, buttocks or other body parts? Touched your body in a sexual way or in a way that made you uncomfortable? By “sexual way” we mean touching you on your genitals, breasts or buttocks. Showed you pictures, magazines, or movies of people or children doing sexual things? Made you take your clothes off when it was not for a medical reason? Opened or took their own clothes off in front of you when they should not have done so? Kiss you when you didn’t want to be kissed? Make you touch their genitals, breasts or buttocks when you didn’t want to? Touch your genitals, breasts or buttocks when you didn’t want them to? Give you money/ things to do sexual things? Involve you in making sexual pictures or videos? Threaten or pressure you to have sex or do sexual things with them? Actually make you have sex with them by threatening or pressuring you, or by making you afraid of what they might do? Make you have sex with them by physically forcing you (have sex with you)?	Coded 1 if answered yes to any of the items; 0 if answered no to all items.
Injury	Have any of the following things ever happened to you as a result of what teachers or adults at your school have done to you, as you told me about above? You felt pain? You had bruising*? You had swelling*? You were bleeding*? You had cuts*? It was difficult to sit down on your buttocks*? It was difficult to walk*? You lost consciousness, even temporarily**? You suffered a dislocated, sprained, fractured or broken bone**? You had any other serious injury**? You had to get medical attention, for example from the health worker or hospital**? You had to stay home from school?	Coded 1 if answered yes to any of the items; 0 if answered no to all items.
*moderate injury		
**severe injury		
Physical violence from school staff	What are the methods of physical discipline you have used with students? Have you ever: Slapped them with a hand on their face or head as punishment? Twisted their ear as punishment? Twisted their arm as punishment? Pulled their hair as punishment? Hit them by throwing an object at them? Hit them with a closed fist? Hit them with a stick? Caned them? Kicked them? Knocked them on the head as punishment? Made them dig, slash a field, or do other labour as punishment? Hit their fingers or hands with an object as punishment? Crushed their fingers or hands as punishment? Made them stand/kneel in a way that hurts to punish them? Made them stay outside for example in the heat or rain to punish them? Burnt them as punishment? Taken their food away as punishment? Forced them to do something that was dangerous? Choked them? Tied them up (with a rope or belt) at school? Tried to cut them purposefully with a sharp object? Made them roll over on the ground until they were dizzy as punishment?	Coded 1 if answered yes to any of the items; 0 if answered no to all items

The International Society for the Prevention of Child Abuse and Neglect Child Abuse Screening Tool-Child Institutional (ICAST-CI) [21] and some items from the WHO Multi Country Study on Women’s Health and Domestic Violence against Women [22] were used to measure experiences of violence. Reliability and construct validity for the ICAST-CI were initially established in 4 countries and the instrument has since been translated into 20 languages and used extensively in multi-country research [21]. Lifetime and past week experience of physical, sexual, emotional violence and injuries were constructed as binary variables (see Table 1).

Symptoms of common childhood mental disorders including depression, anxiety and conduct disorders were measured using the Strengths and Difficulties Questionnaire (SDQ) [23] brief screening instrument which has been used in more than 60 different countries (including several in Africa) and validated in a variety of settings [23]. In our sample reliability for global difficulties scores was Cronbach alpha = 0.70. The global SDQ score was constructed as a categorical variable, with children having ‘high’, ‘medium’ or ‘low’ levels of difficulties relative to their peers. Responses from 20 items were summed to construct this measure and children scoring in the highest decile of the overall distribution were deemed to have ‘high’ difficulties; the next decile to have ‘medium’ difficulties and the remaining 80% to have ‘low’ difficulties [23, 24].

Demographic variables included: age of the child in years, whether or not the child ate three meals versus less than three meals in the past day, whether the child shared a sleeping area with two other children versus less, whether the child shared a sleeping area with an adult versus not, if the child does one-two, or two or more, hours of paid or unpaid work outside of school versus less than 1 hour.

### Analysis

All analyses were conducted using Stata 12.0 [25] and were carried out separately for male and female participants. Data collection was electronic with algorithms designed to eliminate erroneous skips, so levels of missing data were less than 1% for multivariate analysis. Missing data were excluded from analyses involving those variables (pairwise deletion).

We present descriptive statistics on participants’ demographic characteristics and prevalence of different forms of violence by disability status. Comparisons are made using Chi-squared tests for binary variables, Chi-squared tests for trend for categorical variables, or t-tests for continuous variables.

To examine whether factors associated with violence differed between disabled and non-disabled students, we fitted a series of logistic regression models with interaction terms for disability and exposure variables (demographic and mental health difficulties). We created models for emotional violence/neglect in boys and girls, physical violence in boys and girls, and sexual violence in girls. Levels of sexual violence were higher among disabled boys, however overall prevalence was too low (only 9 disabled boys reported sexual violence), to allow us to statistically model predictors. Of all interactions tested, only one was statistically significant, hence we present models examining factors associated with different forms of violence for male and female students, controlling for the main effect of disability.

All analyses account for the sampling scheme employed in the baseline survey—student responses are weighted to account for unequal probabilities of selection for students. Standard errors are adjusted for clustering at the school level using Taylor linearization [26].

## Results

### Characteristics of students

3706 students completed the survey and their characteristics are summarised in Table  2. Of these students, 8.8% of boys and 7.6% of girls reported a disability. Distribution of different forms of disabilities were similar between boys and girls in this sample: 2.8% of all students reported difficulties with sight, 1.4% with hearing, 0.9% with movement, 0.5% with speech, and 3.1% reported an ‘other’ form of disability (students could report more than one form of disability). However, 1.0% of boys but only one girl reported difficulties with speech in our sample. We also asked if students suffered from ‘fits’ (epilepsy) but only two students reported this.

**Table 2:** **Demographic characteristics in students with and without disabilities, full sample**

Characteristic	No disability (n = 3435),%**	Disabled (n = 271),%**	p ^a^
Male	47.5	51.3	
Female	52.5	48.7	0.148
Age			
10 years or less	4.6	3.6	
11 to 14 years	81.7	82.3	
15 or more years	13.7	14.1	0.656
Meals			
1 meal	13.0	13.4	
2 meals	37.9	41.1	
3+ meals	49.1	45.6	0.494
Transport to school			
Other	3.8	8.7	
Walking alone	25.2	26.3	
Walking with others	62.7	48.9	
Board at school	8.2	16.1	0.001
Ever worked for money	33.7	30.7	0.457
Hours worked*			
Less than 1	40.4	45.6	
1-2 hours	43.7	39.0	
More than 2 hours	15.9	15.5	0.286

*Paid or unpaid hours worked per day on average ^a^Chi-squared test or Chi-squared test for trend; **some percentages sum to more than 100% because of rounding error.

Disabled and non-disabled students were demographically similar in most respects. Most students were aged 11-14 years, and less than half of all students reported eating at least 3 meals in the day before the survey, indicating that they were possibly hungry. Around one third of all students had ever worked for money, and about 15% of students reported doing more than 2 hours of paid or unpaid work per day. The only difference in background characteristics between groups was that disabled students were far more likely to board at school than non-disabled students.

### Experiences of violence

Experiences of physical, sexual, emotional violence or neglect are nearly universal among primary school students in this setting, with more than 95% of all students reporting experiences of violence. Patterns of experiences of violence differed for male and female students, and for disabled students (Table 3). Overall, disabled and non-disabled boys reported similar levels of any forms of violence from any perpetrator, however disabled girls were more likely to report violence exposure than non-disabled girls. Disabled girls reported slightly more physical violence overall and nearly twice as much sexual violence versus non-disabled girls. For disabled boys, levels of sexual violence were nearly double those of non-disabled boys, but given the low prevalence of sexual violence in boys overall this difference was not statistically significant. Levels of emotional violence and neglect were similar between disabled and non-disabled boys and girls.

**Table 3:** **Levels of violence, disabled versus non disabled girls and boys**

	Boys (n = 1769)			Girls (n = 1937)		
Characteristic	No disability,%	Disabled,%	P	No disability,%	Disabled,%	P
Any violence from any perpetrator	95.3	96.2	0.644	95.5	99.5	0.009
Physical violence	93.4	95.8	0.379	94.6	99.1	0.010
Sexual violence	3.8	7.1	0.092	12.3	23.6	0.002
Emotional violence and neglect	59.4	63.6	0.400	57.5	65.2	0.140
Violence from school staff	94.1	94.4	0.893	94.4	97.7	0.109
Physical violence	93.3	93.8	0.786	93.9	98.0	0.076
Sexual violence	1.8	2.6	0.654	2.2	3.6	0.361
Emotional violence and neglect	31.9	40.5	0.003	29.4	38.2	0.053
Injury	64.5	70.0	0.208	68.7	73.5	0.140
Severe physical violence	7.0	5.5	0.566	6.8	10.8	0.123
Past week physical violence	53.0	43.8	0.025	51.2	60.5	0.054
Violence from parents/caregivers	13.3	12.4	0.757	32.3	32.2	0.967
Physical violence	10.7	9.1	0.486	28.1	28.6	0.906
Sexual violence^a^	-	-	-	-	-	-
Emotional violence	4.8	4.2	0.760	8.9	9.9	0.760
Violence from female peers	12.0	13.9	0.527	24.7	33.3	0.152
Physical violence	1.7	2.1	0.756	8.7	9.2	0.901
Sexual violence^a^	-	-	-	-	-	-
Emotional violence	10.5	11.3	0.819	19.0	27.5	0.055
Neglect						
Violence from male peers	48.4	54.3	0.293	31.8	34.4	0.568
Physical violence	31.4	36.3	0.360	21.0	22.2	0.784
Sexual violence^a^	1.1	4.5	0.015	3.7	7.8	0.026
Emotional violence	33.8	35.9	0.702	15.6	16.7	0.764
Violence from others	9.4	10.1	0.779	17.8	24.1	0.081
Physical violence^b^	5.4	9.2	0.186	9.6	15.7	0.0002
Sexual violence^c^	0.3	1.7	0.003	0.1	17.0	0.0001
Emotional violence	9.7	10.2	0.832	15.9	22.0	0.089

^a^Not estimated (too few cases reported) ^b^for females, nearly all others for physical violence are other relatives (12.6% of disabled girls report physical violence by relative versus 4.6% non disabled, p = 0.006) ^c^for females, nearly all ‘others’ for sexual violence are reported as ‘other’—ie not boyfriend, girlfriend, other relative or would rather not say who (14.4% of disabled girls report sexual violence by others verses 6.4% of non-disabled girls); for males, nearly all others are other relatives (1.1% versus 0 non disabled).

### Perpetrators

The patterns of who perpetrated violence also differed for disabled and non-disabled students, and again by sex (Table 3). School staff were less likely to be physically violent towards disabled boys in the past week, but more likely to be emotionally violent or neglectful. For girls, school staff were also more likely to be emotionally violent or neglectful, but also more likely to have used physical violence in the past week. In both disabled girls and boys, levels of severe physical violence and injury from school staff were not statistically different from non-disabled peers.

Both male and female disabled and non-disabled students reported similar levels of violence from caregivers. We also considered violence from male and female peers. Disabled and non-disabled boys reported similar levels of violence from male and female peers, but disabled boys reported more than four times as much sexual violence from male peers versus non-disabled boys. Disabled girls also reported similar levels of violence from male and female peers, but disabled girls were more than twice as likely to report sexual violence from male peers versus non-disabled girls.

From all other perpetrators, disabled and non-disabled boys reported similar levels of violence, with the exception of sexual violence. Disabled boys were 6 times more likely to report sexual violence from ‘others’, versus non-disabled boys. For boys, the main perpetrators of this violence were ‘other relatives’, not parents or caregivers. Disabled girls were at increased risk of physical, sexual and to a lesser extent, emotional violence from other perpetrators. The main ‘other’ perpetrators of physical violence against disabled girls were ‘other relatives’. The main ‘other’ perpetrators of sexual violence against disabled girls were ‘others’—that is, not boyfriends, girlfriends, parents or caregivers, school staff, other relatives or someone they would rather not say (leaving mainly strangers and acquaintances in the community).

### Do factors associated with increased risk of violence differ between disabled and non-disabled students?

Interaction terms were not statistically significant (data not shown), suggesting that factors associated with increased risk of violence are similar for disabled and non-disabled students. Disability itself is associated with increased risk of sexual violence in girls (Table 4).

**Table 4:** **Predictors of different forms of violence in girls and boys**

Characteristic	Emotional violence/neglect versus no emotional violence/neglect		Sexual violence versus no sexual violence		Physical Violence from non-school staff versus no violence from non-school staff		Past week physical violence from school staff versus no past week violence from school staff		Any of these forms of violence versus none	
	**aOR** ^**a**^ **(95% CI)**	**p**	**aOR** ^**a**^ **(95% CI)**	**p**	**aOR** ^**a**^ **(95% CI)**	**p**	**aOR** ^**a**^ **(95% CI)**	**p**	**aOR** ^**a**^ **(95% CI)**	**p**
Girls (n = 1920)										
Age (years, continuous)	0.99 (0.91-1.09)	0.889	1.31 (1.16-1.50)	<0.001	0.86 (0.78-0.94)	0.001	0.91 (0.86-0.96)	0.002	0.90 (0.76-1.06)	0.188
Ate at least 3 meals yesterday	0.93 (0.77-1.13)	0.486	0.83 (0.58-1.18)	0.284	1.12 (0.86-1.47)	0.394	0.81 (0.66-0.99)	0.043	1.03 (0.78-1.36)	0.820
Shares a sleeping area with at least 2 other children versus less	0.78 (0.60-1.02)	0.065	0.72 (0.56-0.92)	0.01	0.88 (0.75-1.03)	0.113	1.04 (0.80-1.37)	0.748	0.74 (0.58-0.93)	0.011
Shares a sleeping area with any adults versus none	0.96 (0.81-1.13)	0.598	0.98 (0.77-1.25)	0.855	1.08 (0.79-1.46)	0.630	1.14 (0.95-1.35)	0.153	1.14 (0.87-1.50)	0.335
Works less than 1 hour per day	1		1		1		1		1	
Works 1-2 hours per day	1.28 (0.99-1.64)	0.055	1.07 (0.77-1.53)	0.685	1.39 (1.02-1.89)	0.037	1.24 (1.02-1.51)	0.030	1.35 (0.92-1.99)	0.125
Works more than 2 hours per day	2.69 (1.80-4.03)	<0.001	1.56 (0.87-2.73)	0.120	1.53 (0.94-2.48)	0.086	1.91 (1.13-3.26)	0.018	3.72 (1.79-7.75)	0.001
Disability	1.36 (0.86-2.13)	0.179	2.15 (1.33-3.48)	0.002	1.26 (0.80-1.99)	0.309	1.46 (1.00-2.12)	0.047	1.52 (0.68-3.39)	0.298
Low mental health difficulties	1		1		1		1		1	
Medium mental health difficulties	2.30 (1.66-3.19)	0.001	1.97(1.35-2.88)	0.001	1.38 (0.92-2.06)	0.113	1.38 (1.04-1.83)	0.025	2.28 (1.52-3.41)	<0.001
High mental health difficulties	2.92 (2.02-4.24)	0.001	2.18 (1.31-3.61)	0.003	1.62 (1.08-2.41)	0.020	2.36 (1.58-3.54)	<0.001	2.95 (1.46-5.94)	0.003
Boys (n = 1759)										
Age (years, continuous)	0.98 (0.90-1.07)	0.624			0.85 (0.80-0.90)	<0.001	0.90 (0.82-0.98)	0.018	0.86 (0.77-0.96)	0.010
Ate at least 3 meals yesterday	0.87 (0.71-1.08)	0.200			1.06 (0.79-1.44)	0.675	0.94 (0.74-1.19)	0.586	1.12 (0.78-1.60)	0.524
Shares a sleeping area with at least 2 other children versus less	0.72 (0.58-0.90)	0.005			0.94 (1.79-1.12)	0.493	0.84 (0.69-1.03)	0.090	0.86 (0.68-1.10)	0.224
Shares a sleeping area with any adults versus none	0.98 (0.81-1.18)	0.812			1.18 (0.85-1.65)	0.317	0.92 (0.68-1.24)	0.558	1.11 (0.80-1.53)	0.523
Works less than 1 hour per day	1				1		1		1	
Works 1-2 hours per day	1.49 (1.14-1.95)	0.005			1.55 (1.16-2.08)	0.004	1.41 (1.11-1.80)	0.006	1.73 (1.27-2.36)	0.001
Works more than 2 hours per day	1.54 (0.99-2.42)	0.056			1.62 (0.99-2.67)	0.056	1.23 (0.94-1.61)	0.132	1.59 (0.97-2.63)	0.067
Disability	1.08 (0.74-1.56)	0.672			1.17 (0.80-1.69)	0.409	0.62 (0.48-0.81)	0.001	1.01 (0.62-1.63)	0.972
Low mental health difficulties	1				1		1		1	
Medium mental health difficulties	2.21 (1.47-3.34)	<0.001			1.37 (1.01-1.87)	0.045	2.02 (1.47-2.80)	<0.001	2.55 (1.51-4.28)	0.001
High mental health difficulties	3.05 (1.86-5.01)	<0.001			1.84 (1.25-2.71)	0.003	2.30 (1.67-3.16)	<0.001	3.63 (1.81-7.28)	0.001

^a^Adjusted for all other variables in model.

Disability was also associated with increased risk of past week physical violence from school staff for girls, but decreased risk for boys. Being disabled was not statistically significantly associated with emotional violence or physical violence from others besides school staff in boys or girls. Having medium or high levels of mental health difficulties was consistently associated with all forms of violence exposure in both boys and girls. Working outside of school for 1-2 hours or 2 or more hours per day was also associated with several forms of violence experience in both boys and girls, although the association was more consistent across different forms of violence in boys. Sharing a sleeping area with 2 or more children also was associated with decreased risk of emotional and sexual violence in girls, and emotional violence in boys. Other factors were not consistently associated with violence exposure in girls and boys.

## Discussion

Disabled girls show higher levels of victimisation from a variety of perpetrators than their non-disabled peers. They are twice as likely as their non-disabled peers to have experienced sexual violence. Disabled boys are also at increased risk of some forms of violence relative to non-disabled boys. The school environment is a main venue at which violence is occurring, with disabled boys and girls both reporting more emotional violence and neglect from school staff, sexual violence from male peers, and disabled girls reporting more emotional violence from female peers. For girls, disability is associated with increased risk of past week physical violence from school staff, but for boys, being disabled is associated with decreased risk of past week physical violence from school staff. Risk factors for violence experience in disabled and non-disabled students are similar: younger age, working outside the home and having symptoms of common mental disorders are associated with increased violence exposure.

### Other literature

Our findings support the results of multi-country qualitative research in sub-Saharan Africa, which suggested a greater risk of violence against children with disabilities in all countries as well as some variation in types of violence experienced across different disability types. This multi-country research also found that disabled girls were more likely to be subjected to sexual violence [7], similar to our results. Whilst some qualitative evidence highlights that schools are a key setting for the perpetration of violence in LMICs, our study is one of the first studies to provide rigorous population-based epidemiological data on different forms of violence experienced by children with disabilities who attend schools in such settings.

Further research is needed to fully understand why disabled children, and girls in particular, are at increased risk. Some studies in the region have shown that girls with disabilities may face higher levels of violence because of being less able to defend themselves or seek help [12]. Stigma associated with disability, attitudes and traditional beliefs about disabilities, social isolation and the view that children with disabilities are often perceived as unworthy of dignity and respect, combined with traditional gender norms in many LMIC settings may also explain why children with disabilities are at greater risk of violence [27].

Further research is also needed to understand how boarding at school may interact with risk. In our study, disabled students were far more likely to board at school. Although parents and caregivers may actually see boarding as a mechanism to protect children from violence, for example, by reducing their exposure to violence as they journey to school, clearly this was not the case. Qualitative research may provide some insight into pathways of risk.

### Strengths and limitations

Our study is cross-sectional, and causal inferences should not be drawn from our findings. We did not survey children who were not attending school, consequently our findings should not be considered representative of disabled children who are not attending school. Available evidence highlights poor access and retention in school for children with disabilities [1], and that children with more severe disabilities are likely to be excluded from school. In resource constrained contexts, parents may not perceive as many longer term economic benefits from schooling for their disabled versus non-disabled children, and may be less likely to send disabled children to school. Lack of physical access to schools is often a major barrier to inclusion for children with physical disabilities, and may also explain the limited reporting of mobility-related challenges.

Often research including disabled children is limited by small sample sizes, making it difficult to do sex-specific analyses. We have a reasonably large sample size, which helps to look at patterns, but statistical power to look at the relative contributions of different determinants separately by sex is still limited.

Our survey contained one question assessing difficulties with functioning in multiple domains to measure disability. More detailed measures may have detected more cases of disability. We also recognise that shame or stigma associated with disability could have led to under-reporting of disability [28, 29]. Furthermore, evidence suggests that children with disabilities are often socially isolated, have limited support networks and few friends, which can make reporting about violence more difficult [6]. We did not ask about level of severity of disabilities and it is possible that children experiencing more severe difficulties were more likely to self-identify and that milder difficulties were under-reported. Further, we did not ask children to self-report intellectual disability. This may explain the higher percentage of children under ‘other’. Some studies suggest that intellectual disability may place young people at particular risk [13], and it would be interesting to explore this in future research.

Our measure of emotional violence may also have underestimated the prevalence of discrimination and ‘bullying’ related to children’s disability status. The measure included items related to maltreatment because of skin colour, gender, religion, tribe and health problems, but we did not specifically mention disability. A measure including more specific items may have detected higher levels of emotional violence in disabled children.

### Implications

Uganda has a favourable policy climate, which reflects the government’s commitment to address issues of accessibility, participation, capacity-building, awareness raising, care and support of disabled children. Despite these positive policy initiatives, our findings highlight the need for school-based interventions to reduce violence against all children, paying particular attention to the increased risks of violence associated with disability. Although there are an increasing number of interventions to address school-based violence in low income settings, there is limited evidence on their efficacy. In contrast, there is more rigorous evidence available for some school-based interventions to bullying between students, with school-wide programs more successful at reducing bullying versus curriculum-based programs [30]. There is a clear need to develop and test interventions to reduce violence from school staff towards students—to our knowledge, the Good Schools Study is the only rigorous study underway to evaluate an intervention aimed at reducing staff violence. There is also a clear need to develop effective programs to address ‘dating violence’ between peers [31]. New interventions should be inclusive and accessible to disabled students. This is particularly true of interventions to address sexual violence—although disabled students form less than 10% of the school population in our sample, they have about double the rates of sexual violence. Interventions to reduce sexual violence must address the additional vulnerability faced by disabled students.

## Conclusions

In Uganda, disabled children attending school are at increased risk of most forms of violence relative to their non-disabled peers. Schools are a main risk environment, but also provide an opportunity to deliver interventions to reduce violence. Further research is needed to understand the patterns and potential to prevent violence against children with disabilities inside and outside of school in low income countries, particularly sexual violence against girls.

## Abbreviations

GSS: Good Schools Study
LMICs: Low and middle-income countries
ICAST-CI: International Society for the Prevention of Child Abuse and Neglect Child Abuse Screening Tool-Child Institutional
WHO: World Health Organization
SDQ: Strengths and Difficulties Questionnaire.
